# Ultrastructural and Proteomic Analyses Revealed the Mechanism by Which Foliar Spraying of Se Nanoparticles Alleviated the Toxicity of Microplastics in *Pistia stratiotes* L.

**DOI:** 10.3390/toxics13110938

**Published:** 2025-10-30

**Authors:** Sixi Zhu, Haobin Yang, Yutian Lv, Suxia Sun, Wei Zhao, Zhongbing Chen

**Affiliations:** 1 The Karst Environmental Geological Hazard Prevention of Key Laboratory of State Ethnic Affairs Commission, College of Eco-Environment Engineering, Guizhou Minzu University, Guiyang 550025, China; 2 Guiyang Institute of Information Science and Technology, Guiyang 550025, China; 3 Department of Applied Ecology, Faculty of Environmental Sciences, Czech University of Life Sciences Prague, Kamýcka 129, 16500 Praha-Suchdol, Czech Republic

**Keywords:** nanoplastic, proteomics, molecular mechanism, selenium nanoparticles

## Abstract

The uptake and accumulation of nanoplastics by plants have emerged as a major research focus. Exogenous selenium nanoparticles (SeNPs) are widely used to mitigate the toxicity of abiotic stresses, such as nanoplastics (NPs) and polyethylene (PE—NPs) nanoplastics, and represent a feasible strategy to enhance plant performance. However, the molecular mechanisms by which SeNPs alleviate the phytotoxicity of microplastics and nanoplastics remain poorly defined. To address this gap, we used *Pistia stratiotes* L. (*P. stratiotes*) as a model and silicon dioxide nanoparticles (SiO_2_NPs) as a comparator, integrating physiological assays, ultrastructural observations, and proteomic analyses. We found that NP stress caused ultrastructural damage in root tips, exacerbated oxidative stress, and intensified membrane lipid peroxidation. SeNPs treatment significantly mitigated NP-induced oxidative injury and metabolic suppression. Compared to the NPs group, SeNPs increased T-AOC by 38.2% while reducing MDA and ·OH by 33.3% and 89.6%, respectively. Antioxidant enzymes were also elevated, with CAT and POD rising by 47.1% and 39.2%. SeNPs further enhanced the photosynthetic capacity and osmotic adjustment, reflected by increases in chlorophyll a, chlorophyll b, and soluble sugar by 49.7%, 43.8%, and 27.0%, respectively. In contrast, proline decreased by 17.4%, indicating stress alleviation rather than an osmotic compensation response. Overall, SeNPs outperformed SiO_2_NPs. These results indicate that SeNPs broadly strengthen anti-oxidative defenses and metabolic regulation in *P. stratiotes*, effectively alleviating NP-induced oxidative damage. Proteomics further showed that SeNPs specifically activated the MAPK signaling cascade, phenylpropanoid biosynthesis, and energy metabolic pathways, enhancing cell-wall lignification to improve the mechanical barrier and limiting NPs translocation via a phytochelatin-mediated vacuolar sequestration mechanism. SiO_2_NPs produced similar but weaker alleviative effects. Collectively, these findings elucidate the molecular basis by which SeNPs mitigate NPs’ phytotoxicity and provide a theoretical foundation and practical outlook for using nanomaterials to enhance phytoremediation in aquatic systems.

## 1. Introduction

According to statistics, 79% of the world’s plastics (up to 65.6 billion tons) are in the natural environment and landfills [1]. NPs are plastic particles < 5 mm in size and are recognized as emerging contaminants with a high hazard potential, low detectability under routine monitoring, and long-term environmental persistence [2]. It is a serious pollution problem in terrestrial and aquatic ecosystems [3]. The primary sources of NPs in the environment are agricultural plastic sheets, tire wear particles, fertilizer application, and plastic film use. These materials can release many NPs smaller than 100 nm [4], which enter the ecosystem via land runoff, wastewater discharge, and atmospheric deposition [5]. It has been fully confirmed that microplastics severely negatively impact aquatic ecosystems [6]. Microplastic pollution in water will directly inhibit the germination of plant seeds [7], reduce plant biomass, and inhibit the photosynthesis of photosynthetic pigments [8]. It also leads to the massive accumulation of hydroxyl radical (·OH) and superoxide anion (O_2_^−^·) [9]. Moreover, both terrestrial and aquatic plants can adsorb and accumulate these highly mobile NPs in the environment, such as Potamogeton crispus, Arabidopsis thaliana [10], Oryza sativa [11], and Vallisneria densesettulata [12]. The absorbed NPs can inhibit plant growth by affecting nutrient cycling, homeostasis, oxidative stress, and photosynthesis [13]. Recent reviews have revealed that intracellular nanoplastics can traverse cellular membranes and induce oxidative stress, protein homeostasis disruption, mitochondrial dysfunction, and DNA damage at the organelle level, indicating their potential genotoxicity and neurotoxicity risks [14].

Selenium (Se) is one of the most important micronutrients in animal, plant, and human life activities [15]. Low Se content in crops could lead to 1 billion people suffering from Se deficiency [16]. This highlights the importance of Se in enhancing biological activities. Compared with inorganic Se, SeNPs show excellent biological activity and efficient utilization at low concentrations, significantly reducing toxicity induced by environmental abiotic stresses, and exhibit favorable dispersibility and antibacterial activity [17,18]. Previous studies have shown that SeNPs have great potential in reducing the absorption of microplastics by plants and can regulate plants’ intrinsic anti-oxidative stress capacity [3]. Our studies have found that SE-like compounds can directly remove the excess hydroxyl radical (·OH) and superoxide anion (O_2_^−^·) in plants and convert O_2_^−^· to hydrogen peroxide without involving superoxide dismutase (SOD) [19]. Se is also an important component of glutathione peroxidase (GSH-Px), which can promote GSH biosynthesis by regulating sulfur metabolism in plants [20]. Therefore, an in-depth analysis of the molecular mechanism of SeNPs in plants can clarify the molecular process of SeNPs regulating the response of aquatic plants to NP stress in detail. Research within the crop system of rice, employing a comprehensive suite of evidence from biochemical, cytological, physiological, and transcriptomic studies, elucidates a significant alleviation of nanoplastic stress coupled with a restructuring of defense-associated pathways. This work provides a valuable blueprint for exploring nanotechnological approaches to mitigate NP stress [21].

Our understanding of plants’ intrinsic mechanisms from physiological and cytological perspectives remains limited; however, the application of multi-omics approaches promises to disentangle the complex response networks among free-floating aquatic macrophytes [22]. Existing studies indicate that cellular energy metabolism, cellular activities, hormone signaling, and antioxidant responses are orchestrated within plant metabolic response pathways and are regulated by the expression of enzymatic proteins [23]. However, integrative frameworks that combine multi-omics, ultrastructural analyses, and molecular biology to reveal the underlying molecular mechanisms in aquatic plants are still lacking [24]. Proteomics, a core technique widely applied in studies of abiotic stress, enables the systematic identification of key functional proteins and signaling pathways, thereby illuminating the molecular regulatory networks underlying stress tolerance [25]. A recent proteomic study showed that selenite supplementation markedly up-regulated proteins involved in cell-wall metabolism, phytochelatin biosynthesis, and stress signal transduction, thereby promoting the sequestration of environmental toxicants within plant roots [26]. Although omics-based studies have examined selenium in single-chemical contexts, the proteomic mechanisms by which the foliar application of SeNPs regulates cellular responses in *P. stratiotes* under NPs exposure remain insufficiently characterized.

In freshwater ecosystems, free-floating aquatic macrophytes constitute core plant communities highly sensitive to water pollution; their submerged root systems are pivotal for sensing, responding to, and sequestering environmental NPs [27]. *P. stratiotes* is a free-floating aquatic plant widely distributed across latitude bands 10–25° and 23.5–40° in both hemispheres; it forms exceptionally dense root mats, grows and propagates rapidly, and exhibits high sequestration potential [22]. In this study, we hypothesize that, under NP exposure, foliar SeNPs maintain cellular homeostasis by modulating physiological responses and the expression of functional proteins, thereby enhancing tolerance in *P. stratiotes*. To test this hypothesis, we integrated physiological measurements, ultrastructural observations (SEM/LSCM), and proteomic profiling to evaluate the mitigating effects of SeNPs on NP stress systematically. We aimed to elucidate the regulatory mechanisms mediated by SeNPs at the both physiological and molecular levels and to identify key functional proteins and metabolic pathways associated with stress tolerance. Our findings provide novel multi-omics insights into aquatic plant responses to microplastics following foliar SeNPs application, thereby informing the assessment of tolerance mechanisms and ecological risks posed by emerging contaminants.

## 2. Materials and Methods

### 2.1. Experimental Plant Materials

Stock (parent) plants of *P. stratiotes* were collected from Bijie City, Guizhou Province, China (27°17′9.1″ N, 105°17′11.8″ E) and from Weining Caohai, Guizhou Province (26°49′–26°53′ N, 104°12′–104°18′ E). Sampling was conducted in mid-March 2024, corresponding with the local early spring active growth period. After collection, the stock plants were maintained in one-quarter-strength Hoagland nutrient solution, which was renewed every five days [22]. Three consecutive acclimation cycles (15 d per cycle) were conducted under controlled indoor greenhouse conditions. Following acclimation, experimental seedlings were obtained by vegetative (asexual) propagation from the maternal *P. stratiotes* plants [24].

### 2.2. Experimental Materials and Experimental Design

Polystyrene nanoplastic particles (NPs; nominal diameter 50 nm; stock concentration 2.5% (*w*/*v*), i.e., 25 mg·mL^−1^; and aqueous dispersion) were purchased from Jiangsu Zhichuan Intelligent Technology (Suzhou) Co., Ltd., Suzhou, Jiangsu, China. (Cat. No. 2024-277-352-Fpad1050ps; Lot No. 20240518-fps). Working solutions were prepared by diluting the stock according to the proportions described in this study. SeNPs (105,062.1 mg/mL, diameter: 40–60 nm) and SiO_2_NPs (100,361, particle size 20 nm, and purity 99 wt%) were purchased from Jiangsu XFNANO Materials Tech Co., Ltd., Nanjing, Jiangsu, China. [18]. *P. stratiotes* seedlings (106°37′36 ″E′26°22′ 26″ N) precultured for 15 days from Caohai, Weining, were returned to the Molecular Biology Laboratory of Guizhou University for Nationality. The surface of the seedlings was sterilized with 5% hydrogen peroxide (H_2_O_2_) solution for 5 min. The rinses were then repeated more than five times with sterile deionized water. Treated *P. stratiotes* seedlings were cultured in 15 × 16 × 20 cylindrical glass containers with 1.5 L of deionized water and maintained in 1/4 concentration Hoagland nutrient solution for *P. stratiotes* growth conditions. The light intensity of the greenhouse was 176 μmol m^2^ s^−1^, the light/dark cycle was 14/10 h, the relative humidity was 70% ± 5%, and the temperature was constant at 25 °C [28]. At treatment initiation, each pot containing 1.5 L of quarter-strength Hoagland solution was spiked with NPs to a nominal concentration of 1 mg L^−1^. During subsequent routine solution renewals, NPs were re-dosed at 1 mg L^−1^ in proportion to the volume replaced to maintain the nominal concentration (i.e., 1.5 mg per complete 1.5 L renewal per pot) [8].

### 2.3. Dose Selection and Environmental Relevance

CK (vehicle control): KCl (1.2 mg L^−1^), NaHCO_3_ (13.0 mg L^−1^), MgSO_4_·7H_2_O (24.7 mg L^−1^), and CaCl_2_·2H_2_O (58.5 mg L^−1^); NPs (1 mg L^−1^); SeNPs (1 mg L^−1^); and SiO_2_NPs (20 mg L^−1^). To match ionic strength and pH across treatments, all formulations used the same vehicle as CK. SeNPs and SiO_2_NPs were prepared from 10 mg L^−1^ and 200 mg L^−1^ stock dispersions, respectively, and diluted 1:10 immediately before application to reach the working concentrations. During the seedling and early vegetative stages, sprays were applied at a fixed nozzle–leaf distance and constant pressure to minimize variability in leaf uptake. Spraying was performed six times at the seedling stage and six times at the early vegetative stage; each application delivered 20 mL per pot (total 240 mL per pot). Accordingly, the cumulative foliar dose was 0.24 mg plant^−1^ for SeNPs and 4.8 mg plant^−1^ for SiO_2_NPs [8,18]. Prior to each foliar application, the nanoparticle suspensions were sonicated for 30 min at ~20 °C to improve dispersion. This pretreatment is commonly used in nanomaterial exposure studies to promote stable suspensions [29]. Each plant was grown individually in a separate pot from sowing. From the six biological replicates, three plants with comparable growth were selected for phenotypic trait analyses [22].

### 2.4. Physiological and Bioinformatic Analyses

#### 2.4.1. Measurement of Physiological Traits

The fresh weight of plants before and after treatment was measured using the growth rate of biomass (GRB) to determine the effect of NP stress on plant toxicology. At the same time, the improvement of plant growth by SeNPs and SiO_2_NPs was determined. The growth of *P. stratiotes* was recorded every 5 days. Photos were taken and recorded. After treatment, the roots and leaves of *P. stratiotes* were collected, wrapped in tin foil, rapidly frozen in liquid nitrogen, and transferred to a −80 °C refrigerator for storage for physiological, cytological, and proteomic analyses [19,28]. The tip tissues of *P. stratiotes*’ newly formed roots were collected and immediately immersed in electron microscopy fixative (2.5% glutaraldehyde (C_5_H_8_O_2_),100 mM phosphate, and pH 7.0–7.5). The assay was subsequently analyzed under a scanning electron microscope (JSM-840, JEOL, Tokyo, Japan) [24]. The following parameters were quantified using commercial assay kits (MLBIO, Shanghai Enzyme-Linked Biotechnology Co., Ltd., Shanghai, China) according to the manufacturer’s instructions: total antioxidant capacity (T-AOC), malondialdehyde (MDA), hydroxyl radicals (·OH), chlorophyll a, chlorophyll b, catalase (CAT) activity, peroxidase (POD) activity, proline (Pro), soluble sugars, ascorbic acid (AsA), glutathione (GSH), and total flavonoids [30].

GRB = increased biomass ÷ biomass before treatment × 100%(1)

Increased biomass = biomass after treatment − biomass before treatment(2)

#### 2.4.2. SEM Analysis

The root tips soaked in fixative were rinsed using phosphate buffer (0.1 M) and then dehydrated by an alcohol gradient of 30%, 50%, 70%, 80%, and 100% (twice). Each step lasted for 10 min. Afterward, the samples were dried using an XD-1 carbon dioxide critical point dryer (Eiko, Tokyo, Japan) and sprayed with gold coating using an IB-3 ion gold-plating instrument (Eiko, Tokyo, Japan). The samples were then observed by JSM-840 scanning electron microscope (manufacturer: JEOL Ltd., Tokyo, Japan; sourced from Guangzhou Puchuan Testing Technology Co., Ltd., Guangzhou, China) [24].

#### 2.4.3. LSCM Analysis

To examine intracellular organization of *P. stratiotes* after co-exposure, leaf and root tissues were collected, rinsed with distilled water to remove surface residues, embedded, and cryo-sectioned at 10 µm thickness. Sections were imaged on a LSM 710 confocal laser scanning microscope with ZEN (blue edition) software (manufacturer: Carl Zeiss Microscopy GmbH, Jena, Germany; sourced from Guangzhou Puchuan Testing Technology Co., Ltd., Guangzhou, China). Imaging parameters were excitation 405 nm; emission 542 nm; objective Plan-Apochromat 20×/0.8 (M27); pinhole 2.79 Airy units (≈90 µm); frame size 512 × 512; and pixel size 0.83 µm pixel^−1^. Images were processed in ImageJ, version 1.54p (National Institutes of Health, Bethesda, MD, USA)for thresholding and morphological analysis to assess treatment effects on cellular structure [31].

### 2.5. Proteome Profiling

Fresh leaves were ground, suspended, and centrifuged, and the precipitate was extracted as protein. The protein was digested using a filter-aided sample preparation procedure, and the peptide content was estimated. The searches used a peptide mass tolerance of 20 ppm and a production tolerance of 0.02 Da, resulting in a 5% false discovery rate (FDR). The Gene Ontology (GO) annotation proteome classified the proteins derived from the UniProt-GOA database (http://www.ebi.ac.uk/GOA/, accessed on 16 August 2025). The KEGG database was used to annotate the protein pathway.

### 2.6. Statistical Analysis

All physiological and biochemical parameters were measured in three independent biological replicates. Data are reported as mean ± standard error (SE). For proteomic two-group comparisons, differential protein abundance was evaluated with two-sided Student’s *t*-tests using thresholds of *p* < 0.05 and fold change > 1.5. GO terms, KEGG pathways, and protein domains were tested for enrichment against the background of all identified proteins using two-tailed Fisher’s exact tests. Multiple testing was controlled by the Benjamini–Hochberg false discovery rate (FDR); adjusted *p* ≤ 0.05 was considered significant [28,30]. Charts were drawn using Origin 2021 and Adobe Illustrator. Proteome data were visualized using the online platform (www.majorbio.com) [28].

### 2.7. Research Consent Statement

This study was approved by Caohai National Nature Reserve Administration Committee.

## 3. Results and Discussion

### 3.1. Plant Morphology Under Different Treatments

**Figure 1:**
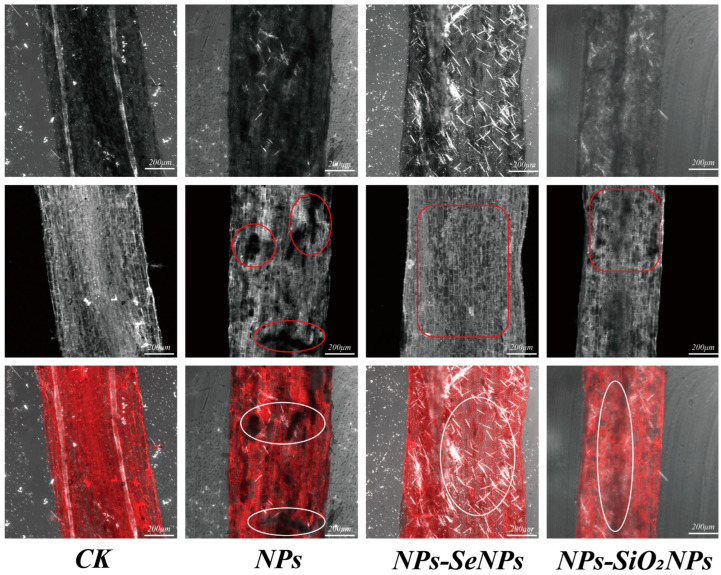
LSCM images of tissues (cross sections) of *P. stratiotes* under different treatments. CK is the control, and NPs (1 mg/L NPs), NPs-SeNPs (1 mg/L NPs; 0.24 mg per plant SeNPs), and NPs-SiO_2_NPs (1 mg/L NPs; 4.8 mg per plant SiO_2_NPs) are the treatments. The SD and the means (n = 3) are represented by the error bars and bars. Different lowercase letters above the bars indicate significant differences at the level of *p* < 0.05.

**Figure 2:**
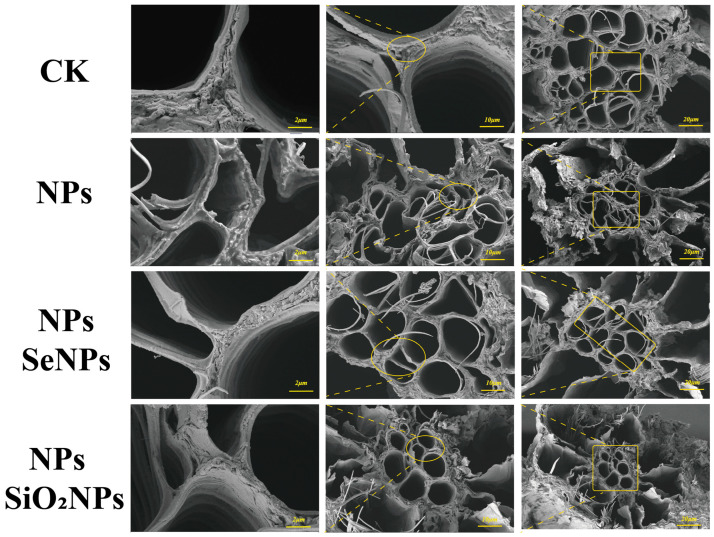
SEM images of tissues (cross sections) of *P. stratiotes* under different treatments. CK is the control, and NPs (1 mg/L NPs), NPs-SeNPs (1 mg/L NPs; 0.24 mg per plant SeNPs), and NPs-SiO_2_NPs (1 mg/L NPs; 4.8 mg per plant SiO_2_NPs) are the treatments. The SD and the means (n = 3) are represented by the error bars and bars.

Using scanning electron microscopy ((SEM) and confocal laser scanning microscopy (LSCM), we verified acropetal (root-to-shoot) transport in *P. stratiotes*, documented NP-induced root damage, and assessed the defense-enhancing effects of nanomaterials (SeNPs and SiO_2_NPs) under NPs stress. In the CK group, root-tip cells showed no evident ultrastructural deformation; under NPs exposure, however, some root cells exhibited necrosis, electron-dense deposits accumulated on both sides of the cell wall, the plasma membrane became distorted, and the overall cellular architecture lost mechanical integrity (Figure 1). Consistent with prior work, nanoplastics readily traverse membrane systems and accumulate in chloroplasts, causing thylakoid disassembly [32]. Our results likewise indicate that NP entry disrupts the root tissue architecture and intracellular homeostasis. By contrast, SeNPs and SiO_2_NPs effectively alleviated NP-induced injury, with SeNPs exerting markedly stronger effects than SiO_2_NPs; exogenous nanoparticles partially protected root-tip tissues, and no obvious surface roughening or deformation was observed [33,34] (Figure 2). In general, the accumulation and translocation of abiotic stressors within plants elicit oxidative stress responses that severely impair normal physiological activity and hinder growth and development [24,35]. Electron-dense deposits and plasma membrane collapse observed in NP-treated roots are characteristic ultrastructural indicators of oxidative injury, a common consequence of abiotic stress [31]. To cope with environmental stress, plants deploy defenses such as cell-wall thickening and vacuolar sequestration [36,37]. The root cell wall is the plant’s primary barrier restricting the entry of external NP stressors. Modulation by trace elements can fortify this barrier, typically by promoting lignin and cellulose deposition, thereby strengthening the plant’s first line of defense [24,38]. In the present study, SEM and LSCM observations provided direct evidence that further corroborates this mechanism of root cell-wall reinforcement.

**Figure 3:**
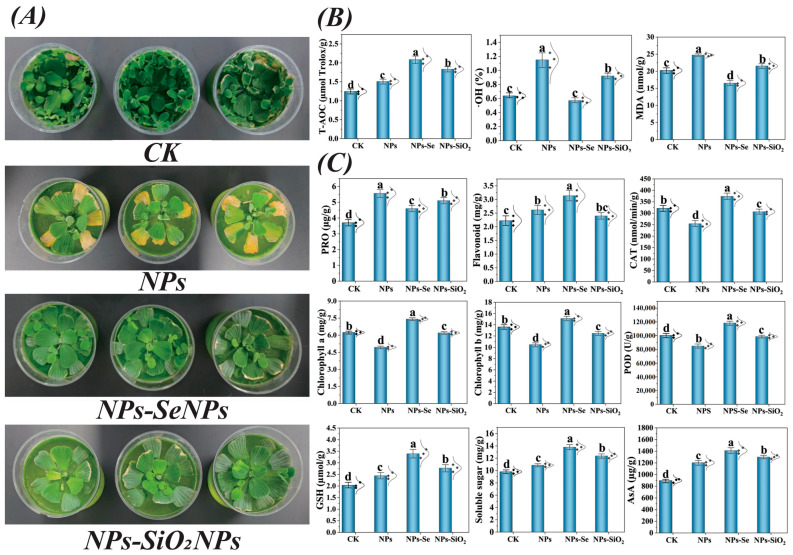
Effects of foliar application of nanomaterials on the growth of Pistia stratiotes under NP stress. (**A**) Comparison of plant growth among different treatments. (**B**) Effects of exogenous nanomaterial application on the total antioxidant capacity (T-AOC), hydroxyl radicals (·OH), and malondialdehyde (MDA) levels of *P. stratiotes* under different stress conditions. (**C**) Effects of exogenous nanomaterial application on P. stratiotes under different stress conditions, including proline (PRO), flavonoids, catalase (CAT), chlorophyll a (Chl a), chlorophyll b (Chl b), peroxidase (POD), glutathione (GSH), soluble sugars, and ascorbic acid (AsA). CK represents the control group; NPs (1 mg·L^−1^ NPs), NPs–SeNPs (1 mg·L^−1^ NPs with 0.24 mg SeNPs per plant), and NPs–SiO_2_NPs (1 mg·L^−1^ NPs with 4.8 mg SiO_2_NPs per plant) are the treatment groups. All data were obtained from three independent biological replicates and are presented as mean ± standard error. Different lowercase letters above the bars indicate significant differences at *p* < 0.05, according to one-way ANOVA followed by Duncan’s multiple range test.

Beyond structural reinforcement, foliar application of exogenous SeNPs significantly reduced the NPs’ burden in both roots and leaves compared with the control. Furthermore, changes in Se content were found during elemental detection, confirming previous findings that both inorganic and nano-forms of Se can effectively act on plant cells to reduce the accumulation of environmental stress factors, thereby improving plant stress resistance [26,39]. We considered that the apparent decline in measured NPs content after foliar Se application could arise from Se—NPs interactions—such as adsorption, complexation, co-aggregation, or co-carriage—that modify their in planta fate and the detectable analytical signal [40]. Prior studies have shown that interactions between Se and other elements can reduce the accumulation of toxic metals in plants and remodel the associated metabolic pathways [33]. Consistent with this, nanoplastics have been observed to co-associate with metalloids (e.g., arsenic, As) to form co-contaminant assemblages, thereby altering their distribution patterns within plant tissues [41]. Based on the indirect evidence presented above, we consider the interaction with SeNPs to be a plausible explanation for the apparent reduction in NPs content. Alternatively, biodilution resulting from rapid plant growth may also account for this observation [42]. Furthermore, it is plausible that the foliar application of SeNPs mediates its effects through the redistribution of NPs into the cell wall and vacuoles, as well as through the activation of key functional proteins involved in transport and lignin biosynthesis, thereby reinforcing the mechanical strength of the cell wall [43,44] (Figure 3A).

### 3.2. SeNPs Enhance the Antioxidant Enzyme Activities of P. stratiotes

To test our hypothesis, we quantified antioxidant enzyme activities and bioregulatory metabolites in *P. stratiotes* under NP stress. Relative to the NPs group, exogenous foliar SeNPs treatment decreased MDA and ·OH levels by—33.3%% and 89.6%, respectively, indicating reduced membrane lipid peroxidation [45] (Figure 3B). Its mechanism may be related to the reduction in oxidative stress in *P. stratiotes* cells by promoting the ascorbic acid–glutathione (AsA-GSH) cycle and glutamine biosynthesis pathway. Under stress conditions, the balance of reactive oxygen species (ROS) is an important way for plants to resist stress. The activities of antioxidant enzymes, such as POD and GSH, were increased in *P. stratiotes* after the exogenous leaf spraying of SeNPs.

Our study showed that exogenous foliar spraying of SeNPs significantly enhanced the GSH content in *P. stratiotes*, which aligns with our previous study assumption (Figure 3C). GSH is a key substrate in plant complexin (PC) biosynthesis and is important for removing abiotic ions from the environment [46]. A recent study showed that SeNPs can promote the absorption and transport of NPs in plants, and the mechanism may be related to the biosynthesis of plant complexin, in which glutathione also plays an important role [47]. Selenocysteine is the primary active substance of glutathione peroxidase (GSH-Px), and its formation is affected by the direct reaction between GSH and Se elements. Se is an essential component of GSH-Px; it may consequently enhance GSH biosynthesis by interacting with sulfur metabolic pathways [20,48]. Given the chemical similarity between Se and sulfur S and the role of Se as an integral component of glutathione peroxidases (GPX), we hypothesize that Se can enhance GSH biosynthesis via sulfur metabolic pathways. In turn, this would alleviate oxidative stress in plants under abiotic challenges.

### 3.3. Protein Expression Profiling and Functional Enrichment Analysis

**Figure 4:**
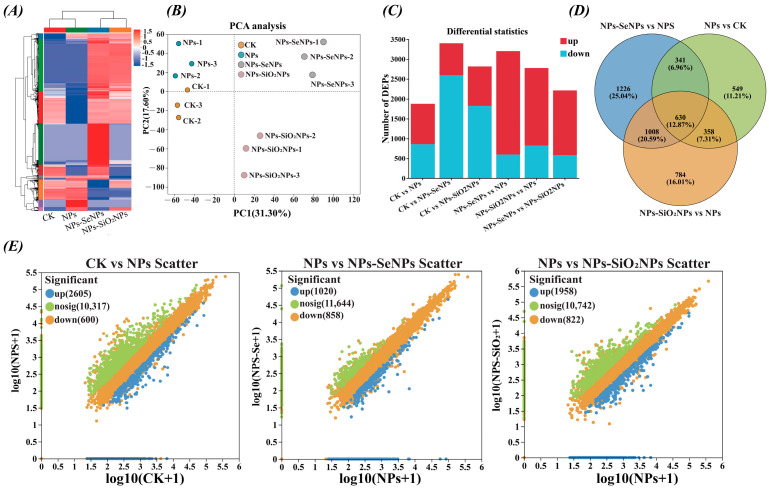
Distribution of differentially expressed proteins (DEPs). (**A**) Cluster analysis of differentially expressed proteins among treatment groups. (**B**) principal component analysis between treatment groups. (**C**) The number of up- and down-regulated proteins in plants under different treatments. (**D**) Venn diagram of DEPs expression in plants under different treatments. (**E**) Volcano plot of plant DEPs up-regulation and down-regulation under different treatments. CK is the control, and NPs (1 mg/L NPs), NPs-SeNPs (1 mg/L NPs; 0.24 mg per plant SeNPs), and NPs-SiO_2_NPs (1 mg/L NPs; 4.8 mg per plant SiO_2_NPs) are the treatments. The SD and the means (n = 3) are represented by the error bars and bars. Different lowercase letters above the bars indicate significant differences at the level of *p* < 0.05.

We further profiled protein expression in *P. stratiotes* to elucidate how selenium mediates mitigation under NP stress. Hierarchical clustering and principal component analysis (PCA) of the proteome profiles revealed clear separation among the four treatments: CK, NPs, NPs-SeNPs, and NPs-SiO_2_NPs. Along PC1, CK and NPs samples exhibited negative scores, whereas NPs-SeNPs and NPs-SiO_2_NPs exhibited positive scores. The sample dendrogram in the heat map mirrored the PCA, indicating that nanomaterials induced global expression states distinct from both CK and NPs (Figure 4A,B). Venn diagrams and differential protein statistics indicated that NPs alone predominantly down-regulated proteins (1226 proteins). With SeNPs and SiO_2_NPs, the proportion of proteins up-regulated relative to the NPs baseline rose to 25% and 20%, respectively (Figure 4C,D; Table S1). Trend scatter plots further revealed an overall shift from the NPs’ condition toward the NPs-SeNPs’ and NPs-SiO_2_NPs’ conditions, indicating nanomaterial-dependent proteomic remodeling (Figure 4E). These results suggest that nanomaterials may induce directional reprogramming of gene expression.

**Figure 5:**
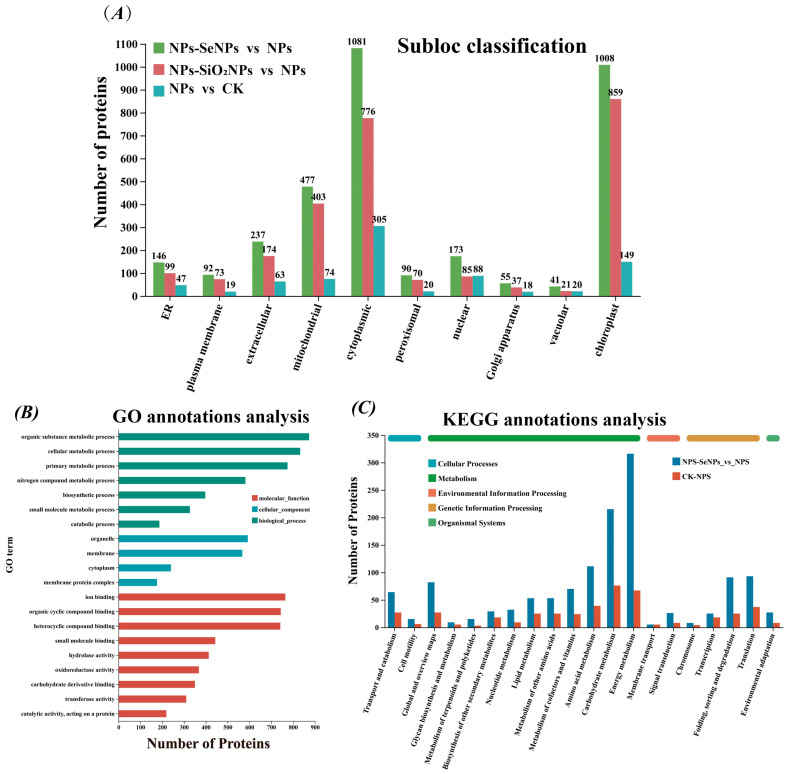
Detailed annotation information of plant functional proteins. (**A**) Subcellular localization (Subloc annotation). (**B**) GO annotation. (**C**) Kyoto Encyclopedia of Genes and Genomes (KEGG annotation). CK is the control, and NPs (1 mg/L NPs), NPs-SeNPs (1 mg/L NPs; 0.24 mg per plant SeNPs), and NPs-SiO_2_NPs (1 mg/L NPs; 4.8 mg per plant SiO_2_NPs) are the treatments. The SD and the means (n = 3) are represented by the error bars and bars. Different lowercase letters above the bars indicate significant differences at the level of *p* < 0.05.

**Figure 6:**
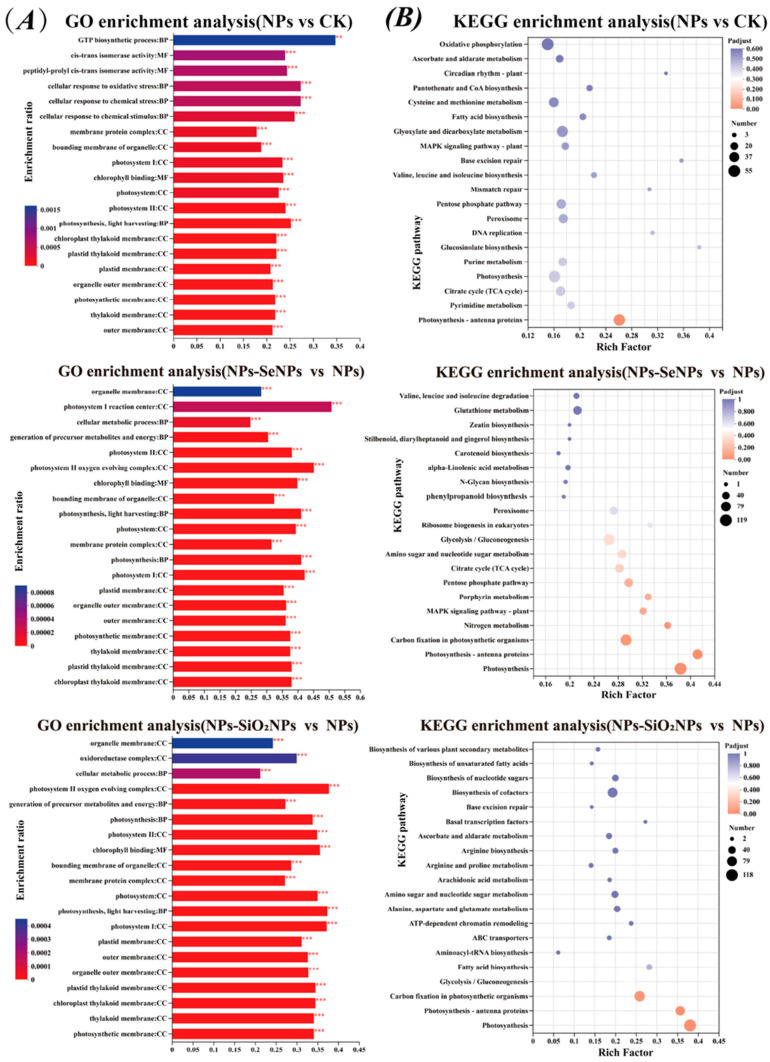
GO and KEGG pathway enrichment analyses of hub DEPs. (**A**) GO functional pathway enrichment analysis of DEPs in CK (control group without treatment), NPs, NPs-SeNPs, and NPs-SiO_2_NPs treatments. DEPs can be divided into three ontologies (Biological Processes (BPs), Molecular Functions (MFs), and cellular components (CCs)). Asterisks indicate significance of GO-term enrichment: ** *p* < 0.01, *** *p* < 0.001. (**B**) The significance of up-regulated and down-regulated DEPs on KEGG for the first 20 pathways in CK, NPs, NPs-SeNPs, and NPs-SiO_2_NPs treatments. The x-axis represents the rich factor and the y-axis represents the name of the path. Bubble size indicates the amount of DEPs involved, and bubble color indicates the degree of pathway enrichment. (For interpretation of the references to color in this figure legend, the reader is referred to the Web version of this article).

Subcellular localization predictions indicated that DEPs localized primarily in the cytosol and chloroplasts, followed by mitochondria and the plasma membrane, which are key compartments for energy conversion, redox homeostasis, and stress perception and signaling. (Figure 5A; Table S2). GO enrichment analysis revealed significant overrepresentation of the terms’ organic substance metabolic process and nitrogen compound metabolic process in the Biological Process category, and oxidoreductase activity and hydrolase activity in the Molecular Function category (Figure 5B; Table S3). KEGG pathway analysis and classification highlighted significant enrichment in fundamental metabolic processes—such as carbohydrate, amino acid, and energy metabolism—as well as in pathways related to protein folding, sorting, degradation, and signal transduction (Figure 5C; Table S4). Notably, the number of differentially expressed proteins (DEPs) in the NPs-SeNPs vs. NPs comparison exceeded that in NPs vs. CK, suggesting that SeNPs not only partially reverse stress-induced injury but also elicit additional tolerance mechanisms. GO term refinement revealed distinct patterns across the comparisons: the NPs vs. CK group showed enrichment in stress response and photosynthetic membrane terms. In contrast, the NPs-SeNPs vs. NPs group shifted towards energy generation, including terms related to the photosystem complex and ATP synthesis (coupled proton transport). The NPs-SiO_2_NPs vs. NPs group exhibited a similar, though weaker, trend (Figure 6A; Table S3). Relative to NPs, foliar SeNPs treatment yielded 626 DEPs (Tables S1 and S5). Enrichment analysis of the top 20 pathways in the NPs-SeNPs versus NPs comparison revealed significant overrepresentation of the MAPK signaling pathway, α-linolenic acid metabolism, phenylalanine metabolism, amino sugar and nucleotide sugar metabolism, galactose metabolism, and starch and sucrose metabolism (Figure 6B). Among these, the plant hormone signaling pathway is pivotal for rapid oxidative stress responses and the initiation of defense programs, playing a central role in the adaptation to abiotic [23]. Collectively, these pathways coordinate a comprehensive stress response that spans signal perception and transduction, energy provision, and the production of secondary metabolites, thereby providing the metabolic resources and signaling framework that underpin plant defense.

### 3.4. Protein Regulation and Conformational Activation of Key Defense Mechanisms

Building on the proteomic data, we further delineated the key defense mechanisms activated by SeNPs. At the level of signal transduction, SeNPs treatment was associated with activation of the MAPK cascade. Our proteomic data indicated that SeNPs specifically activated the MAPK signaling cascade. Structurally, the plant MAPK module—comprising MAPKKK, MAPKK, and MAPK—features a conserved bilobal kinase domain in its MAPK component. The activity of this domain is regulated by the phosphorylation of the activation loop (A-loop) and depends on critical HRD and DFG motifs. Phosphorylation of the TEY motif within the A-loop promotes formation of the Lys–Glu salt bridge in the αC-helix and alignment of the regulatory spine (R-spine), thereby stabilizing the active kinase conformation and enhancing ATP and substrate binding [49]. In light of our data, we posit the presence of a comparable conformational control mechanism; confirmation will need structural or site-directed mutagenesis experiments (Table S6). Mechanistically, treatment with SeNPs may activate MAPK signaling by promoting the phosphorylation of MAPKs and the activation of their upstream kinases, including MAPKKs and MAPKKKs. This phosphorylation stabilizes the active kinase conformation, enhances catalytic throughput, and amplifies downstream signaling. Consequently, these events accelerate the initiation of root defense responses and expedite the propagation of signals. Moreover, the MAPK cascade functions as an integrative hub for multiple hormone cues, initiating at the receptors or co-receptors and relaying information via sequential phosphorylation to nuclear transcription factors, thereby enabling continuous signal transduction from the membrane to the nucleus. Accordingly, we infer that SeNPs treatment may further potentiate defense responses by increasing hormone receptor sensitivity and modulating downstream components.

**Figure 7:**
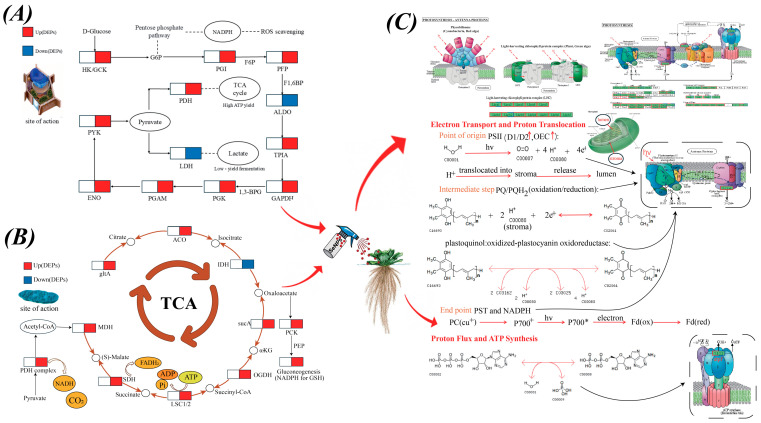
Exogenous spraying of nanomaterials induced key defense mechanisms of *Pistia stratiotes* in response to nanoplastic (NP) stress. (**A**) Schematic representation of the glycolysis pathway. (**B**) Differentially expressed proteins (DEPs) involved in the tricarboxylic acid (TCA) cycle. (**C**) Diagram of the photosynthetic electron transport chain. Red and blue boxes indicate commonly up-regulated and down-regulated DEPs, respectively.

To counteract the toxicity of environmental stressors such as NPs, plants initiate early stress responses by generating reactive ROS, which modulate the activity and expression of protein kinases and phosphatases [50]. ROS trigger MAPK cascades and coordinate with calcium and hormone signaling to induce APX, CAT, and GR, thereby limiting oxidative damage [51]. However, excessive ROS following acute stress damages organelles and perturbs normal cellular functions. This imbalance in self-regulation is a key driver of toxicity, necessitating the timely removal of oxidants through enzymatic and non-enzymatic mechanisms [52]. Accordingly, at the antioxidant-detoxification level, SeNPs treatment promoted the upregulation of antioxidant enzymes and proteins involved in GSH metabolism. At the energetic level, glycolysis and the pentose phosphate pathway (PPP) jointly supply ATP and reduce power. ATP supports GSH biosynthesis via ATP-dependent γ-ECS and GSHS, whereas NADPH drives GSSG reduction and peroxidase-mediated detoxification (Figure 7A). This interpretation is consistent with increased cellular GSH and with the upregulation of glutathione S-transferase omega-1 (GSTO1) and sulfite oxidase (SUOX), which together provide a biochemical basis for antioxidant and detoxification responses. Moreover, interplay and competition between selenium and sulfur metabolic routes may enhance selenoenzyme activity and GSH biosynthesis, thereby accelerating ROS scavenging and reducing oxidative burden [26].

The transport–detoxification system likewise depends critically on the cellular energy metabolism. When NADPH and ATP are sufficiently supplied, the expression of key enzymes throughout PC biosynthesis and subsequent vacuolar sequestration is markedly up-regulated, substantially increasing pathway flux and enhancing cellular homeostasis and stress tolerance. Specifically, the mitochondrial TCA cycle and oxidative phosphorylation supply ATP to power ABC transporters and support GSH and PC biosynthesis; in parallel, the chloroplastic photosynthetic electron–transport chain replenishes ATP via photophosphorylation, while ferredoxin–NADP^+^ reductase (FNR) catalyzes the electron transfer from reduced ferredoxin (Fd) to NADP^+^ to produce NADPH (Figure 7B,C). With adequate NADPH and ATP, the GSH and PC pathway not only scavenges reactive ROS and chelates xenobiotics but also furnishes the substrates and energy required for ABCC-type transporter-mediated efflux, culminating in vacuolar sequestration and closure of the detoxification loop. Supported by ATP and NADPH jointly provided by mitochondria and chloroplasts—and given that the initial steps of GSH biosynthesis occur in the chloroplast and cytosol—we infer that flux through the GSH/phytochelatin (PC) detoxification pathway is enhanced [53]. GSH is the biosynthetic precursor of phytochelatins (PCs). Consistent with findings by Song et al., we infer that, in our system, greater reducing power and energy supply—together with increased GSH availability—enhance PC biosynthesis and the subsequent ABCC-mediated (ATP-binding cassette subfamily C) vacuolar sequestration, thereby lowering the cytosolic exposure to toxic species [54]. Regarding SeNPs, accumulating evidence suggests their primary effects arise from S/Se metabolic crosstalk and an overall improvement in redox homeostasis. These changes increase cysteine and GSH pools and enhance the activities of glucose-6-phosphate dehydrogenase (G6PDH), glutathione reductase (GR), and glutathione peroxidase (GPX), thereby indirectly promoting phytochelatin (PC) biosynthesis and increasing flux through the PC-based detoxification pathway [55].

At the structural defense level, treatment with SeNPs significantly induced changes in the expression of various protein kinases and metabolic enzymes in *P. stratiotes*, collectively enhancing cell-wall construction and physical resistance. The expression levels of serine–threonine protein kinase, mitogen-activated protein kinase A (MAPK A), and pelle-like serine–threonine protein kinase were significantly altered. Among these, serine/threonine protein kinases are involved in multiple physiological processes central to cell-wall construction. They participate in the biosynthesis of phenylpropanoid, lignin, pectin, and hemicellulose, and play a crucial role in pectin degradation, enhancing cell-wall mechanical strength and forming plant defense barriers [56]. Becker et al. (2015) demonstrated that the cell-wall integrity (CWI) MAPK cascade in the fungus Epichloë festucae is essential for hyphal network formation and the maintenance of restricted growth during symbiosis with Lolium perenne [57]. Although derived from a fungal system, their findings provide a mechanistic framework for understanding the MAPK-mediated regulation of cell-wall integrity and polarized growth. This concept is highly relevant to models of plant cell-wall remodeling. In plants, pelle-like serine and threonine protein kinases perceive exogenous elicitors such as AVR-Pik via non-catalytic domains, directly activating immune responses [58]. Subsequently, carbon flux is redirected toward the phenylpropanoid–lignin biosynthesis pathway. Under SeNPs treatment, key enzymes in this pathway, such as 4-Coumarate-CoA ligase (4CL, EC 6.2.1.12), are up-regulated. Functioning as a central hub in this pathway, 4CL catalyzes the ATP-dependent ligation of hydroxycinnamic acids to CoA, thereby generating acyl-CoA esters and allocating metabolic flux into multiple branching routes [39]. This process is supported by a dedicated energy metabolism network, where ATP drives the 4CL-catalyzed reaction and downstream polymer assembly, while NADPH fuels the reductive steps catalyzed by cinnamoyl-CoA reductase (CCR) and cinnamyl alcohol dehydrogenase (CAD) (Figure 7A–C). Taken together, foliar SeNPs treatment remodels the protein expression network to coordinately activate multiple defense layers, including signal transduction, antioxidant detoxification, and cell-wall fortification, thereby enhancing the overall tolerance of *P. stratiotes* to NP stress. This study advances the proteomic understanding of stress tolerance in aquatic plants and highlights the greater application potential of SeNPs relative to SiO_2_NPs.

## 4. Conclusions

This study investigated the alleviative effects of foliar selenium nanoparticle (SeNP) application on Pistia stratiotes under nanoplastic (NP) stress by integrating physiological indicators, ultrastructural observations, and proteomic analysis. Our results demonstrate that SeNPs treatment effectively mitigated NP-induced oxidative damage, as evidenced by the enhanced total antioxidant capacity and significant reductions in malondialdehyde (MDA) and hydroxyl radical (·OH) levels. Concurrently, SeNPs boosted the activities of antioxidant enzymes, such as catalase and peroxidase, and regulated the metabolism of non-enzymatic antioxidants, collectively improving the plant’s overall antioxidant defense system. Proteomic analysis further revealed that the protective mechanism of SeNPs likely involves the activation of the MAPK signaling pathway, phenylpropanoid biosynthesis, and energy metabolism. These activated pathways appear to regulate key physiological processes, including cell-wall fortification, redox homeostasis, and vacuolar sequestration, thereby enhancing plant tolerance to NP stress. Compared to silicon dioxide nanoparticles (SiO_2_NPs), SeNPs exhibited superior potential in mitigating NP phytotoxicity.

However, this study has several limitations. The nanoparticle characterization was confined to supplier-provided nominal size, lacking critical data on hydrodynamic size, zeta potential, stability, aggregation state, surface coating, and elemental purity in the exposure media. This gap constrains the interpretation of reproducibility and bioavailability. The controlled greenhouse conditions differ from natural aquatic environments, limiting the direct ecological extrapolation of our findings. Furthermore, the study utilized polystyrene NPs, which do not represent the diversity of plastic types and complex co-contamination scenarios in the environment. The precise interaction mechanisms between SeNPs and NPs within plant tissues remain incompletely elucidated, and the proteomic findings primarily provide correlative evidence. Future work will involve the systematic characterization of nanoparticles in the actual exposure media using TEM, DLS, and EDS/ICP-MS. Including inorganic selenium controls will help distinguish the specific effects of the nanoscale form from general selenium biochemistry. Follow-up studies will assess the safety and remediation potential of SeNPs in real aquatic ecosystems and explore their synergistic effects with other nanomaterials or organic remediation agents. These results would provide a scientific basis for environmental health assessment of new pollutants and environmental protection, which is significant for protecting fragile aquatic ecosystems. It will also provide new insights for effective phytoremediation of NPs-contaminated water.

## Data Availability

The raw data supporting the conclusions of this article will be made available by the authors on request.

## Supplementary Materials

The following supporting information can be downloaded at: https://www.mdpi.com/article/10.3390/toxics13110938/s1, Figure S1: Laser scanning confocal microscopy imaging and analysis; Table S1: Top thirty up-down DEPs statistics protein difference map; Table S2: Subcellular statistical table; Table S3: GO enrichment analysis statistical table all; Table S4: KEGG Enrichment Analysis Statistical Table; Table S5: Overview Table of Protein Function Annotation; Table S6: Top thirty up-down DEPs statistics protein structure diagram.

## References

[1] Geyer R., Jambeck J.R., Law K.L. (2017). Production, Use, and Fate of All Plastics Ever Made. Sci. Adv..

[2] Rillig M.C., Lehmann A. (2020). Microplastics in Terrestrial Ecosystems. Science.

[3] Sun Y.Z., Ji J.H., Tao J.G., Yang Y.Y., Wu D., Han L.F., Li S., Wang J. (2023). Current Advances in Interactions between Microplastics and Dissolved Organic Matter in Aquatic and Terrestrial Ecosystems. TrAC Trends Anal. Chem..

[4] He P., Chen L., Shao L., Zhang H., Lü F. (2019). Municipal Solid Waste (MSW) Landfill: A Source of Microplastics?—Evidence of Microplastics in Landfill Leachate. Water Res..

[5] Carr S.A., Liu J., Tesoro A.G. (2016). Transport and Fate of Microplastic Particles in Wastewater Treatment Plants. Water Res..

[6] Vivekanand A.C., Mohapatra S., Tyagi V.K. (2021). Microplastics in aquatic environment: Challenges and perspectives. Chemosphere.

[7] Shi R., Liu W., Lian Y., Wang Q., Zeb A., Tang J. (2022). Phytotoxicity of polystyrene, polyethylene, and polypropylene microplastics on tomato (*Lycopersicon esculentum* L.). J. Environ. Manag..

[8] Lian J., Liu W., Sun Y., Men S., Wu J., Zeb A., Yang T., Ma L.Q., Zhou Q. (2022). Nanotoxicological effects and transcriptome mechanisms of wheat (*Triticum aestivum* L.) under stress of polystyrene nanoplastics. J. Hazard. Mater..

[9] Zhuang H., Qin M., Liu B., Li R., Li Z. (2023). Combination of transcriptomics, metabolomics, and physiological traits reveals the effects of polystyrene microplastics on photosynthesis, carbon, and nitrogen metabolism in cucumber (*Cucumis sativus* L.). Plant Physiol. Biochem..

[10] Sun X., Yuan X., Jia Y., Feng L., Zhu F., Dong S., Liu J., Kong X., Tian H., Duan J. (2020). Differentially Charged Nanoplastics Demonstrate Distinct Accumulation in *Arabidopsis thaliana*. Nat. Nanotechnol..

[11] Zhou C., Lu C., Mai L., Bao L., Liu L., Zeng E. (2021). Response of rice (*Oryza sativa* L.) roots to nanoplastic treatment at the seedling stage. J. Hazard. Mater..

[12] Tang N., Li X., Gao X., Liu X., Xing W. (2022). The adsorption of arsenic on micro- and nano-plastics intensifies the toxic effect on submerged macrophytes. Environ. Pollut..

[13] Zhang S., Wang H., Liu M., Yu H., Peng J., Cao X., Wang C., Liu R., Kamali M., Qu J. (2022). Press Perturbations of Microplastics and Antibiotics on Freshwater Micro-Ecosystem: Case Study for the Ecological Restoration of Submerged Plants. Water Res..

[14] Casella C., Ballaz S.J. (2024). Genotoxic and Neurotoxic Potential of Intracellular Nanoplastics: A Review. J. Appl. Toxicol..

[15] Reich H.J., Hondal R.J. (2016). Why nature chose selenium. ACS Chem. Biol..

[16] Tolu J., Bouchet S., Helfenstein J., Hausheer O., Chekifi S., Frossard E., Tamburini F., Chadwick O.A., Winkel L.H.E. (2022). Understanding soil selenium accumulation and bioavailability through size-resolved and elemental characterization of soil extracts. Nat. Commun..

[17] Cheng B., Wang C., Chen F., Yue L., Cao X., Liu X., Yao Y., Wang Z., Xing B. (2022). Multiomics understanding of improved quality in cherry radish (*Raphanus sativus* L. var. *Radculus pers*) after foliar application of selenium nanomaterials. Sci. Total Environ..

[18] Wang M., Wang Y.X., Ge C.H., Jing F., Wu S., Li H.B., Zhou D.M. (2023). Foliar selenium nanoparticles application promotes the growth of maize (*Zea mays* L.) seedlings by regulating carbon, nitrogen, and oxidative stress metabolism. Sci. Hortic..

[19] Zhu S., Sun S., Zhao W., Yang X., Mao H., Sheng L., Chen Z. (2024). Utilizing transcriptomics and proteomics to unravel key genes and proteins of *Oryza sativa* seedlings mediated by selenium in response to cadmium stress. BMC Plant Biol..

[20] Schiavon M., Pilon-Smits E.A.H. (2017). The Fascinating Facets of Plant Selenium Accumulation—Biochemistry, Physiology, Evolution, and Ecology. New Phytol..

[21] Jin J., Ghouri F., Xia W., Wang J., Shahid M.Q. (2025). Alleviation of Nanoplastic Stress in Rice: Evidence from Biochemical, Cytological, Physiological, and Transcriptome Analysis. J. Agric. Food Chem..

[22] Zhao W., Chen Z.B., Yang X.Q., Sheng L.Y., Mao H., Zhu S.X. (2023). Integrated transcriptomics and metabolomics reveal key metabolic pathway responses in *Pistia stratiotes* under Cd stress. J. Hazard. Mater..

[23] Chen H., Jin J., Hu S., Shen L., Zhang P., Li Z., Fang Z., Liu H. (2023). Metabolomics and proteomics reveal the toxicological mechanisms of florfenicol stress on wheat (*Triticum aestivum* L.) seedlings. J. Hazard. Mater..

[24] Li X., Hu N., Li Y., Tang H., Huang X., Yang T., Xu J. (2024). Integrated ultrastructural, physiological, transcriptomic, and metabolomic analysis uncovers the mechanisms by which nicotinamide alleviates cadmium toxicity in *Pistia stratiotes* L.. J. Hazard. Mater..

[25] Zhao Y., Gong J., Shi R., Wu Z., Liu S., Chen S., Tao Y., Li S., Tian J. (2025). Application of proteomics in investigating the responses of plant to abiotic stresses. Planta.

[26] Yu Y., Wang Q., Wan Y., Huang Q., Li H. (2023). Transcriptome analysis reveals different mechanisms of selenite and selenate regulation of cadmium translocation in *Brassica rapa*. J. Hazard. Mater..

[27] Yuan W., Xu E.G., Li L., Zhou A., Peijnenburg W.J.G.M., Grossart H.-P., Liu W., Yang Y. (2023). Tracing and trapping micro- and nanoplastics: Untapped mitigation potential of aquatic plants?. Water Res..

[28] Zhu S., Sun S., Zhao W., Yang X., Chen Z., Mao H., Sheng L. (2024). Comprehensive physiology and proteomics analysis revealed the resistance mechanism of rice (*Oryza sativa* L.) to cadmium stress. Ecotoxicol. Environ. Saf..

[29] Eitzen L., Ruhl A.S., Jekel M. (2020). Particle Size and Pre-Treatment Effects on Polystyrene Microplastic Settlement in Water: Implications for Environmental Behavior and Ecotoxicological Tests. Water.

[30] Sixi Z., Sun S., Zhao W., Yang X., Mao H., Sheng L. (2024). Comprehensive physiology and proteomics analysis revealed the molecular toxicological mechanism of Se stress on indica and japonica rice. Chemosphere.

[31] Zhang Y., Yang S., Zeng Y., Chen Y., Liu H., Yan X., Pu S. (2023). A new quantitative insight: Interaction of polyethylene microplastics with soil–microbiome–crop. J. Hazard. Mater..

[32] Xu L., Liu C., Ren Y., Huang Y., Liu Y., Feng S., Zhong X., Fu D., Zhou X., Wang J. (2024). Nanoplastic toxicity induces metabolic shifts in Populus × euramericana cv. ‘74/76′ revealed by multi-omics analysis. J. Hazard. Mater..

[33] Wang M., Li H., Dang F., Cheng B., Cheng C., Ge C., Zhou D. (2024). Common metabolism and transcription responses of low-cadmium-accumulative wheat (*Triticum aestivum* L.) cultivars sprayed with nano-selenium. Sci. Total. Environ..

[34] Guo X., Luo J., Du Y., Li J., Liu Y., Liang Y., Li T. (2021). Coordination between root cell wall thickening and pectin modification is involved in cadmium accumulation in *Sedum alfredii*. Environ. Pollut..

[35] Wang J., Chen X., Chu S., You Y., Chi Y., Wang R., Yang X., Hayat K., Zhang D., Zhou P. (2022). Comparative cytology combined with transcriptomic and metabolomic analyses of *Solanum nigrum* L. in response to Cd toxicity. J. Hazard. Mater..

[36] Krämer U. (2010). Metal hyperaccumulation in plants. Annu. Rev. Plant Biol..

[37] Yadav V., Arif N., Kovac J., Singh V.P., Tripathi D.K., Chauhan D.K., Vaculik M. (2021). Structural modifications of plant organs and tissues by metals and metalloids in the environment: A review. Plant Physiol. Biochem..

[38] Zhu C.Q., Cao X.C., Zhu L.F., Hu W.J., Hu A.Y., Bai Z.G., Zhong C., Sun L.M., Liang Q.D., Huang J. (2018). Ammonium mitigates Cd toxicity in rice (*Oryza sativa*) via putrescine-dependent alterations of cell wall composition. Plant Physiol. Biochem..

[39] Di X., Jing R., Qin X., Wei Y., Liang X., Wang L., Xu Y., Sun Y., Huang Q. (2023). Transcriptome analysis reveals the molecular mechanism of different forms of selenium in reducing cadmium uptake and accumulation in wheat seedlings. Chemosphere.

[40] Agarwal S., Kumari S., Singh N., Khan S. (2023). Fate of plastic nanoparticles (PNPs) in soil and plant systems: Current status & research gaps. J. Hazard. Mater. Adv..

[41] Tang N., Huang W., Li X., Gao X., Liu X., Wang L., Xing W. (2024). Drilling into the physiology, transcriptomics, and metabolomics to enhance insight on *Vallisneria denseserrulata* responses to nanoplastics and metalloid co-stress. J. Clean. Prod..

[42] Wang M., Mu C., Lin X., Ma W., Wu H., Si D., Ge C., Cheng C., Zhao L., Li H. (2024). Foliar Application of Nanoparticles Reduced Cadmium Content in Wheat (*Triticum aestivum* L.) Grains via Long-Distance “Leaf–Root–Microorganism” Regulation. Environ. Sci. Technol..

[43] Wan Y., Wang K., Liu Z., Yu Y., Wang Q., Li H. (2019). Effect of selenium on the subcellular distribution of cadmium and oxidative stress induced by cadmium in rice (*Oryza sativa* L.). Environ. Sci. Pollut. Res..

[44] Cui J., Liu T., Li Y., Li F. (2018). Selenium Reduces Cadmium Uptake into Rice Suspension Cells by Regulating the Expression of Lignin Synthesis and Cadmium-Related Genes. Sci. Total. Environ..

[45] Baryla A., Laborde C., Montillet J.L., Triantaphylidès C., Chagvardieff P. (2000). Evaluation of lipid peroxidation as a toxicity bioassay for plants exposed to copper. Environ. Pollut..

[46] Kang Y., Qin H., Wang G., Lei B., Yang X., Zhong M. (2024). Selenium nanoparticles mitigate cadmium stress in tomato through enhanced accumulation and transport of sulfate/selenite and polyamines. J. Agric. Food Chem..

[47] Wang J., Zhang T., Gao J., Li B., Han L., Ge W., Wang Z. (2024). The accumulation of cadmium and lead in wheat grains is primarily determined by the soil-reducible cadmium level during wheat tillering. Chemosphere.

[48] Handa N., Kohli S.K., Sharma A., Thukral A.K., Bhardwaj R., Abd E.F., Alqarawi A.A., Ahmad P. (2019). Dynamics of antioxidative defence expression, photosynthetic attributes, and secondary metabolites to mitigate chromium toxicity in *Brassica juncea* L. plants. Environ. Exp. Bot..

[49] Taylor S.S., Kornev A.P. (2011). Protein Kinases: Evolution of Dynamic Regulatory Proteins. Trends Biochem. Sci..

[50] Brigelius-Flohé R., Flohé L. (2017). Selenium and Redox Signaling. Arch. Biochem. Biophys..

[51] Lin L., Wu J., Jiang M., Wang Y. (2021). Plant Mitogen-Activated Protein Kinase Cascades in Environmental Stresses. Int. J. Mol. Sci..

[52] Feng R., Wei C., Tu S. (2013). The Roles of Selenium in Protecting Plants against Abiotic Stresses. Environ. Exp. Bot..

[53] Galant A., Preuss M.L., Cameron J.C., Jez J.M. (2011). Plant glutathione biosynthesis: Diversity in biochemical regulation and reaction products. Front. Plant Sci..

[54] Song W.-Y., Park J., Mendoza-Cózatl D.G., Suter-Grotemeyer M., Shim D., Hörtensteiner S., Geisler M., Weder B., Rea P.A., Rentsch D. (2010). Arsenic tolerance in Arabidopsis is mediated by two ABCC-type phytochelatin transporters. Proc. Natl. Acad. Sci. USA.

[55] Abdalla M.A., Lentz C., Mühling K.H. (2022). Crosstalk between selenium and sulfur is associated with changes in primary metabolism in lettuce plants grown under Se and S enrichment. Plants.

[56] Kang L., Wu Y., Jia Y., Chen Z., Kang D., Zhang L., Pan C. (2023). Nano-selenium enhances melon resistance to *Podosphaera xanthii* by enhancing the antioxidant capacity and promoting alterations in the polyamine, phenylpropanoid and hormone signaling pathways. J. Nanobiotechnol..

[57] Becker Y., Eaton C.J., Brasell E., May K.J., Becker M., Hassing B., Cartwright G.M., Reinhold L., Scott B. (2015). The Fungal Cell-Wall Integrity MAPK Cascade Is Crucial for Hyphal Network Formation and Maintenance of Restrictive Growth of *Epichloë festucae* in Symbiosis with *Lolium perenne*. Mol. Plant-Microbe Interact..

[58] Maidment J.H.R., Franceschetti M., Maqbool A., Saitoh H., Jantasuriyarat C., Kamoun S., Terauchi R., Banfield M.J. (2021). Multiple variants of the fungal effector AVR-Pik bind the HMA domain of the rice protein OsHIPP19, providing a foundation to engineer plant defense. J. Biol. Chem..

