# Supplementary material for: Ultrastructural and Proteomic Analyses Revealed the Mechanism by Which Foliar Spraying of Se Nanoparticles Alleviated the Toxicity of Microplastics in Pistia stratiotes L
Source: Toxics. 2025 Oct 30;13(11):938. doi: 10.3390/toxics13110938 (PMC12656514; doi:10.3390/toxics13110938)
Supplement: Supplementary file 1 [file toxics-13-00938-s001.zip › Highlight.pdf]

Highlights

NPS disrupts photosynthesis by altering enzymes required for NADPH and ATP synthesis.

SeNPs promote biosynthesis of photosynthetic and antioxidant proteins in *Pistia stratiotes L.*

SeNPs markedly alleviate NPS-induced oxidative damage in *Pistia stratiotes L.*

SeNPs restore root-cell ultrastructure in *Pistia stratiotes L.* and re-establish cellular homeostasis.
