# Supplementary material for: Ultrastructural and Proteomic Analyses Revealed the Mechanism by Which Foliar Spraying of Se Nanoparticles Alleviated the Toxicity of Microplastics in Pistia stratiotes L
Source: Toxics. 2025 Oct 30;13(11):938. doi: 10.3390/toxics13110938 (PMC12656514; doi:10.3390/toxics13110938)
Supplement: Supplementary file 1 [file toxics-13-00938-s001.zip › Supporting Information.pdf]

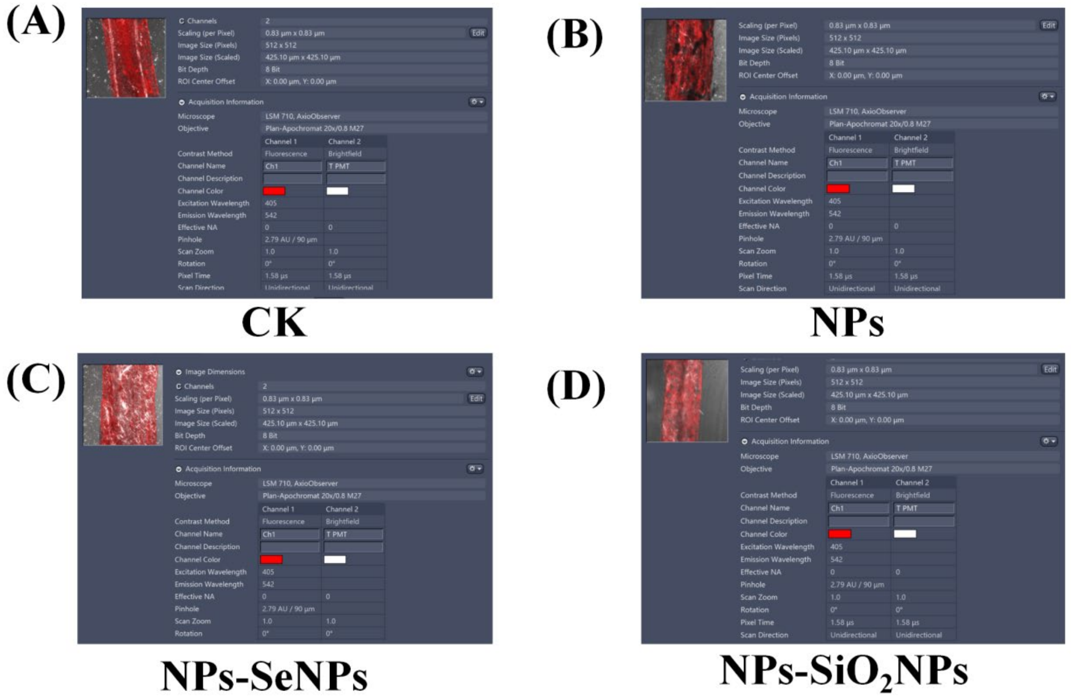

Figure S1. Laser scanning confocal microscopy imaging and analysis. Tissue sections were imaged using a ZEISS LSM 710 confocal microscope controlled by ZEN Blue software. Image acquisition parameters were as follows: excitation wavelength 405 nm, emission wavelength 542 nm; Plan-Apochromat 20×/0.8 M27 objective; pinhole set to 2.79 AU (≈ 90 µm); image frame size 512 × 512 pixels, pixel size 0.83 µm. Acquired images were subsequently subjected to thresholding and morphological analysis using ImageJ (NIH) software.
